# Comparing antiretroviral treatment outcomes between a prospective community-based and hospital-based cohort of HIV patients in rural Uganda

**DOI:** 10.1186/1472-698X-11-S2-S12

**Published:** 2011-11-08

**Authors:** Walter Kipp, Joseph Konde-Lule, Tom Rubaale, Joa Okech-Ojony, Arif Alibhai, Duncan L Saunders

**Affiliations:** 1 School of Public Health, University of Alberta, 3-50 University Terrace, 8303-112 Street, Edmonton, AB, Canada, T6G 2T4; 2 School of Public Health, Makerere University, Kampala, Uganda; 3 District Health Department, Kabarole District Local Government, Fort Portal, Uganda

## Abstract

**Background:**

Improved availability of antiretroviral therapy in sub-Saharan Africa is intended to benefit all eligible HIV-infected patients; however in reality antiretroviral services are mainly offered in urban hospitals. Poor rural patients have difficulty accessing the drugs, making the provision of antiretroviral therapy inequitable. Initial tests of community-based treatment programs in Uganda suggest that home-based treatment of HIV/AIDS may equal hospital-based treatment; however the literature reveals limited experiences with such programs.

**The research:**

This intervention study aimed to; 1) assess the effectiveness of a rural community-based ART program in a subcounty (Rwimi) of Uganda; and 2) compare treatment outcomes and mortality in a rural community-based antiretroviral therapy program with a well-established hospital-based program. Ethics approvals were obtained in Canada and Uganda.

**Results and outcomes:**

Successful treatment outcomes after two years in both the community and hospital cohorts were high. All-cause mortality was similar in both cohorts. However, community-based patients were more likely to achieve viral suppression and had good adherence to treatment. The community-based program was slightly more cost-effective. Per capita costs in both settings were unsustainable, representing more than Uganda’s Primary Health Care Services current expenditures per person per year for all health services. The unpaid community volunteers showed high participation and low attrition rates for the two years that this program was evaluated.

**Challenges and successes:**

Key successes of this study include the demonstration that antiretroviral therapy can be provided in a rural setting, the creation of a research infrastructure and culture within Kabarole’s health system, and the establishment of a research collaboration capable of enriching the global health graduate program at the University of Alberta. Challenging questions about the long-term feasibility and sustainability of a community-based ARV program in Uganda still remain.

**The partnership:**

This project is a continuation of previous successful collaborations between the School of Public Health of Makerere University, the School of Public Health of University of Alberta, the Kabarole District Administration and the Kabarole Research and Resource Center.

## Background

HIV/AIDS is a major global public health problem which particularly affects countries in sub-Saharan Africa [1,2]. Globally, there are 33 million persons living with HIV infection (PLWHIV). Uganda, with one million PLWHIV, ranks as a high HIV prevalence country. The increased availability of antiretroviral treatment (ART) for HIV/AIDS in low income countries has changed the clinical management of PLWHIV and has dramatically increased rates of survival.

Improved availability of ART is intended to benefit all PLWHIV eligible for treatment. However, in reality ART services in developing countries are mainly offered in urban-based hospitals. Thus poor patients living in rural areas cannot access them due to economic and geographic constraints. This inequity in providing ART services is a crucially important issue for health care policy makers and practitioners. In Uganda, the roll-out of ART services to rural areas, where 80% of the population lives, cannot be accomplished by health care workers alone. This is due to the extreme shortage of trained health professionals, which currently remains the biggest single obstacle to the expansion of ART services in Ugandan and all of sub-Saharan Africa. This health personnel shortage is the most acute in rural areas of sub-Saharan Africa. Therefore, the roll-out of ART services to rural populations requires alternative approaches, one of which could be the engagement of communities and community resources.

Various treatment models involving community resources have been developed and tested in Uganda. These include a home-based treatment program in Tororo [3], a home-based treatment program in Jinja [4] and a home-based treatment program in Kampala, which used PLWHIV on treatment as volunteers for outreach [5]. All three programs have shown that home-based treatment is as good as hospital-based treatment. In general, however, the literature reveals that there are limited experiences with community-based ART programs.

## The research

The objectives of our intervention study were: 1) to assess the effectiveness of a rural community-based ART program in Rwimi subcounty, Kabarole district; and 2) to compare the treatment outcomes and mortality in the rural community-based ART program with a well established hospital-based ART program offered at a best practice urban hospital in Kabarole district. The study was undertaken in Rwimi subcounty (population 25 000) which is located in the Kabarole district, western Uganda, 50 km away from the nearest hospital offering ART. This study required less material input (e.g. bicycles vs. motorcycles) than other studies previously done in Uganda which may increase the chance that this model can be replicated using available health sector resources.

The study was a cohort study with a non-randomized intervention design [6]. The study population consisted of a cohort of PLWHIV receiving community-based ART established in a health centre III (run by a clinical officer) in a rural sub-county in Kabarole district and the comparison population consisted of a cohort of PLWHIV receiving ART from the best practice hospital (run by physicians) located in an urban area in the district. In each cohort, all HIV patients presenting themselves to the hospital and the rural clinic for treatment were sequentially recruited after the project commenced. Only those patients were enrolled who were eligible for treatment according to the Ugandan HIV treatment guidelines. Each cohort was followed up for two years to compare mortality and ART outcomes between the cohorts. In the community-based cohort 185 patients were sequentially recruited and in the hospital-based cohort 200 patients were recruited. Several sub-studies were conducted, e.g. gender impact on treatment results, quality of life of community-based patients on treatment, cost-effectiveness of community-based and hospital-based treatment models.

Forty-one lay community volunteers agreed to participate in the pilot and were provided with training on ART. The motivation of the unpaid volunteers was based on an intrinsic desire to support access to treatment for their fellow community members and was reinforced through the recognition and support they received from the health care program and the community. Volunteers were asked to make weekly visits to their patients, during which time they monitored adherence to treatment, through pill counts, as well as assessed the presence of adverse reactions. Volunteers were asked to refer those with clinical problems and/or adverse reactions to ARVs to the clinical officer at the health centre. On a monthly basis the volunteers obtained a supply of ARVs from the local health centre and delivered these to their assigned patients. At the time of recruitment, patients were also asked to identify a family member/friend as their daily treatment supporter to help with the daily intake of the drugs. The first line antiretroviral drugs, provided free of charge by the Ugandan national HIV/AIDS program, consisted of stavudine, lamivudine, and nevirapine.

Outcome measures of the study were: 1) mortality, HIV-1 RNA viral load (VL) per ml and increase in CD4 cell count/µl which were assessed after two years of starting ART. Statistical procedures included descriptive, univariate and multivariate analysis. Logistic regression models assessed the association between successful treatment outcome (VL<400 copies/ml) and clinical and demographic variables. The AIDS-related survival of hospital and community patients was also described. Ethics approval was provided in Canada by the University of Alberta’s Health Research Ethics Board and in Uganda by the School of Public Health, Makerere University, Kampala and the Uganda National Council of Science and Technology, Kampala. Each participant was informed about the study and signed a consent form. Limitations were: 1) the study was not designed with a randomized study design because randomization simply was not feasible; 2) the loss of patients for follow-up, including due to mortality, was relatively high (30% in the community-based cohort vs 29% in the hospital-base cohort).

## Results and outcomes

No patient in the study was reported to have had severe adverse reactions which required a change to second-line treatment. Seventeen patients in the community-based cohort developed a skin rash (6 patients in the hospital-based cohort) and 25 community-based patients reported suffering from mild peripheral neuropathy (50 in hospital-based patients) which did not warrant a change in medication. Successful treatment outcomes after two years in both the community and hospital cohorts were high. However, in the community-based cohort, HIV patients were more likely to achieve viral suppression. CD4+ cell counts increased similarly in both cohorts. All cause mortality was similar in community and hospital patients after the two year time interval. Community-based patients had excellent overall adherence to treatment at rates throughout the study period (adherence was not consistently measured in the hospital cohort). Almost all patients on treatment in the community-based cohort reported a significant increase in their overall quality of life, as measured by a standardized health-related quality of life (HRQL) questionnaire [7].

Women benefited more from the community-based ART program, as more women sought treatment than men (58.3% were females). In addition, in the first six months, women had better treatment outcomes in terms of successful suppression of the VL compared to men [8,9]. However, this difference in the treatment outcome between men and women decreased after two years and was not statistically significant in the longer term. An indirect benefit for women and their partners was that all seven children born to treated women in the community cohort were HIV negative after testing, sparing them and their families the additional burden of caring for an HIV-infected infant.

When program costs were calculated for both, the hospital-based and community-based treatment model, the cost-effectiveness per successful treated HIV patient was slightly better in community-based HIV patients. However, the overall program costs per year per patient in both programs (approximately 100 US$ including the cost of drugs) were at a level that could not be sustained by local Primary Health Care (PHC) Services in Uganda which currently spends on average between 10-20 US$ per person year for all health services. This suggests that the expansion and long-term maintenance of ART programs will require long-term external funding.

The ART treatment model with unpaid volunteers and clinical officers/nurses in charge of serving patients referred by the volunteers has shown to be sustainable for the two year period that this program was evaluated. This is best illustrated by the low attrition of volunteers, as only two volunteers left the program. The recognition efforts by our project and the community were obviously felt by the volunteers to be valuable and very positive. The low attrition and the effective participation of ordinary community members as unpaid volunteers led us believe that this model can be sustained, if the required resources for their supervision, recognition and motivation can be provided. In contrast to popular belief that volunteer programs are cheap, our cost calculations showed that this is not the case, even though the cost-effectiveness per successful treatment was slightly better in the community treatment model compared to the hospital model.

## Challenges and successes

The main success of this study was the demonstration that ART can be provided to rural HIV patients closer to where they live. Our community-based treatment model involved unpaid community volunteers, supported by non-cash incentives, training and supervision and who were connected to a local health centre staffed by clinical officers and nurses. The treatment outcomes of this model were equivalent (and even slightly superior) to the treatment outcomes of ART in the best-practice regional hospital in the district. As ART was delivered through the existing rural Ugandan health care system with minimal additional material and human resources, it can be a feasible model for replication on a larger scale, e.g. district wide. Another success was that a research infrastructure and culture of research has been created amongst the health system leadership in Kabarole district and a cadre of trained and capable research assistants has been established. This has already led to new applied research initiatives of the Kabarole District Health Management Team (DHMT) to generate new information required for better addressing the major health problems in the district such as malaria, tuberculosis, and reproductive health. Finally, the foundation of these collaborations and the field site in Uganda was instrumental in the establishment of the first graduate program in global health in Canada at the University of Alberta in 2005.

Despite these successes, some challenges remain, related to the longer term sustainability of the program. Questions that are raised and need to be answered include:

1) Can the DHMT in Kabarole continue with this program successfully on its own?

2) If this program is to be replicated, what are the factors to be considered to influence successful implementation on a larger district or region-wide scale and which modifications have to be made from the original smaller pilot program?

## The partnership

This project is a continuation of previous successful collaborative HIV/AIDS work in Uganda by the School of Public Health, Makerere University (MU), the School of Public Health, University of Alberta (UofA), the Kabarole Health Department (KHD) and the Kabarole Research and Resource Center (KRC). This partnership started in 1997 between the Department of Public Health Sciences at the UofA and the Department for Epidemiology and Biostatistics, Institute of Public Health, MU. The role of MU and UofA was to collaboratively design the study, apply for funding and supervise the project implementation in Uganda. The KHD’s role was to implement the grant under the supervision from MU and UofA which was somewhat unique to a research project, since the research implementation team usually consists of researchers and not health care workers. We decided to implement this study in the context of a rural health clinic with involvement of the local and district stakeholders in health care. Our hypothesis was that if the research project was already integrated in a practical health care setting with local involvement, it would be much easier for the health care workers to use the research results. Our aim from the beginning of the study was that the intervention, if successful, should be continued as the method of service provision to guarantee future ART to all patients enrolled. With this study design of integrating the research project into the local health services, we felt confident that this project could be more easily handed over with better prospects of successful continuation through the local Primary Health Care services. The special role of the KRC, whose mandate in the district is to plan and deliver on community-based projects for the underserved population, was to support the role of the community volunteers and the community itself in the ownership of the project. This concept of integration distinguishes our research project from many other research projects which don’t strive for a continuation after research funding has ended.

In the past our Ugandan-Canadian collaboration has been very successful in attracting funding from diverse funding agencies (Canadian International Development Agency (CIDA), the International Development and Research Centre (IDRC), the Canadian Institutes of Health Research (CIHR) and the Government of Alberta) with some $3 million obtained in research and development funding. We were diligent from the beginning to establish a multi-disciplinary research team which would enable us to cover a wide range of perspectives in community-based/population-based research initiatives. Disciplines we included were global health, health administration, public health, nursing, epidemiology and biostatistics, clinical (tropical) medicine, social sciences, gender equality, health economy, adult education, communication, etc. We also tried as much as possible to include qualitative and qualitative research methods in our proposals.

The basis for the successful joint work of this partnership has been the mutual respect and trust among the various collaborators which was built over the years through substantive and persistent efforts. Important was, especially at the beginning, the development of personal relationships which was only possible through on site visits and practical collaborative work. Effective and regular communication, which we did often in person and by phone, is a must. It is very difficult in our opinion to establish research collaborations only by electronic communication. If personal relationships have been established, then online communication works much better and is feasible. Our collaboration over the years was very unproblematic with only a few minor incidents. For our volunteers in this project, the liaison between themselves, their communities and an external foreign institution like the UofA was particularly appreciated, which contributed to their high motivation and low drop out rate (only two volunteers have left the program to date after four years). This strong sentiment around the value of external relations was expressed by the volunteers in many informal encounters with us and also in formal research results. Our experience in this and other research/development projects in sub-Saharan Africa has shown us that the continued external links such as ours (which may include only very limited funding) are indeed a crucial ingredient to a sustained partnership and realized to project outcomes. Another way to link developing and developed countries could be the establishment of formal or informal partnerships between Canadian (external) and developing country towns, schools, and other interested groups. There is a huge potential and interest in Canada for this which has not been fully explored and which needs to be further developed and harvested.

This experience leads us believe that an ongoing long term partnership model for development is what is needed. This is in contrast to the development model in many international development agencies which favours a time limited collaboration and a so called “handing over” of project activities to the developing partner without further financial support. It is well known that this short-term model has failed in many development/research projects, since projects “handed over” to the local developing country partner disintegrate very fast and often completely disappear. However, it is obvious that an ongoing partnership between developed and developing country institutions poses several challenges. One is the continued necessity for a research-based organization to raise funds for this type of partnership which absorbs a lot of working capacity and time. In our case we have maintained our collaboration for 14 years which is much beyond the usual duration of development projects funded by bilateral international funding agencies. Another challenge is to tackle research topics of mutual benefit that are directly relevant to services provided at the grassroots level. A third collaborative partnership challenge is always the capacity building component. For us it involved training of local health care workers and local researchers and Canadian graduate students. Over time, more than 30 graduate students from UofA participated in this or other collaborative projects and successfully completed their thesis or their practicum. All of the thesis students have published their study results in respected peer reviewed journals. Many of their findings have been used by the KHD and have influenced service delivery and policy. Some of them are now professionals in development organizations in Canada and other countries.

Finally, based on our success to date in this collaborative project as well as others, we have found academic institutions to be ideal research partners for service delivery agencies in developing countries. With shared goals and mutually desired outcomes, both partners compliment the skill set and contributions of each other to enhance essential services in countries where they are desperately needed. The synergy that is created in this collaborative process helps to overcome all the challenges.

## List of abbreviations used

ART: antiretroviral treatment; CD4: cluster of differentiation 4; DHMT: district health management team; HIV: human immunodeficiency virus; AIDS: acquired immune deficiency syndrome; PHC: primary health care; PLWHIV: persons living with HIV infection; RNA: ribonucleic acid; VL: viral load.

## Competing interests

All authors declare no conflict of interest.

## Authors' contributions

WK was involved in all stages of the study and wrote the article. JKL had major input in the development of the proposal and execution of the study as well as interpretation of study results. He also commented on the draft manuscript. TR was involved in the design of the study and supervised the field work in Uganda. He also provided input into the manuscript and helped to interpret the study results within the context of the Ugandan health care system. JO was part of the proposal writing and supervised the field work. He was involved in the interpretation of the study findings and suggestions on how they could be used for the expansion and scaling-up in Kabarole District. AA was involved in all stages of the proposal development and implementation of the study, supervision of the field work and gave major input into the manuscript and the interpretation of the data. He played a major part in the statistical data analysis.
